# Natural history and neurodevelopmental outcomes in perinatal stress induced hyperinsulinism

**DOI:** 10.3389/fped.2022.999274

**Published:** 2022-10-31

**Authors:** Winnie M. Sigal, Ohoud Alzahrani, Gabriela M. Guadalupe, Herodes Guzman, Jerilynn Radcliffe, Nina H. Thomas, Abbas F. Jawad, Diva D. De Leon

**Affiliations:** ^1^ Division of Endocrinology and Diabetes, Children’s Hospital of Philadelphia, Philadelphia, PA, United States; ^2^ Department of Pediatrics, The Perelman School of Medicine, University of Pennsylvania, Philadelphia, PA, United States; ^3^ Behavioral Neuroscience Core, Center for Human Phenomic Science, The Children’s Hospital of Philadelphia, Philadelphia, PA, United States; ^4^ Division of Developmental and Behavioral Pediatrics, The Children's Hospital of Philadelphia, Philadelphia, PA, United States; ^5^ Division of Child and Adolescent Psychiatry, The Children's Hospital of Philadelphia, Philadelphia, PA, United States; ^6^ Department of Psychiatry, The Perelman School of Medicine, University of Pennsylvania, Philadelphia, PA, United States; ^7^ Department of Pediatrics, Children's Hospital of Philadelphia, Philadelphia, PA, United States; ^8^ Biostatistics Core, Center for Human Phenomic Science, The Children’s Hospital of Philadelphia, Philadelphia, PA, United States

**Keywords:** hypoglycemia, insulin, neonates, brain damage, glucose

## Abstract

**Objective:**

To describe perinatal stress induced hyperinsulinism (PSIHI), determine the prevalence of neurodevelopmental differences, and identify risk factors for poor developmental prognosis.

**Methods:**

Subjects with a history of hyperinsulinism (HI) and perinatal stress and in whom resolution of the HI was demonstrated were included. Medical record review, caregiver interview, and three validated developmental assessments were completed.

**Results:**

Of the 107 subjects (75% male), 36% were born between 32 and 37 weeks. Median age of hypoglycemia presentation was 0 days. Median age at HI diagnosis was 12 days (IQR 13.5). Median length of time for initiation of definitive treatment was 14 days (IQR 14).

Caregiver interviews were completed for 53 of 79 eligible subjects. Developmental concerns were reported by 51%. Neurodevelopmental assessments were completed by caregivers of 37 of the 53 enrolled subjects. The proportion of subjects scoring >1 SD and >2 SD away from the mean in the direction of concern on the major composite scores was significantly greater than in the general population (40.5% vs. 15.8%, *P* ≤ 0.0001 and 18.9% vs. 2.2%, *P* ≤ 0.0001, respectively).

Male sex, small for gestational age status (SGA), and treatment with continuous feeds were associated with assessment scores >1 SD from the mean (*P* < 0.05). SGA and preeclampsia were associated with assessment scores >2 SD from the mean (*P* < 0.05).

**Conclusion:**

While the majority of infants presented with hypoglycemia in the first day of life, diagnosis and treatment occurred 12–14 days later. Children with PSIHI are at high risk of neurodevelopmental deficits and are more likely to perform below average on developmental assessment.

## Introduction

Hyperinsulinism (HI) is the most common cause of persistent hypoglycemia in neonates, infants and children (1). While genetic forms of HI are rare, transient HI associated with perinatal stress is common, and may affect up to 50% of high-risk neonates (2). Despite perinatal stress induced HI (PSIHI)'s relatively high prevalence, the natural history of the disease course is inadequately characterized, with published literature limited to small series using varying definitions of stress induced or transient HI (3–6).

It has been well-established that children with HI are at high risk for poor neurodevelopmental outcomes (7, 8). Persistent hypoglycemia in the neonatal period and infancy, particularly when due to hyperinsulinism, is known to have detrimental effects on the developing brain, leading to permanent brain damage (9). As such, neonatal hypoglycemia due to HI may be one of the most readily preventable causes of neurodevelopmental impairment. However, the true prevalence of these complications in the population of children with neonatal hypoglycemia due to perinatal stress induced HI (PSIHI) is unclear.

Our aims are to characterize a cohort of children with PSIHI, to determine the prevalence of neurodevelopmental differences in this population, and to identify risk factors for poor developmental prognosis.

## Materials and methods

A cross-sectional study was conducted in individuals with perinatal stress induced hyperinsulinism who were diagnosed between 1 January 2013 and 30 September 2018 and received care at the Children's Hospital of Philadelphia (CHOP) Congenital Hyperinsulinism Center. Subjects were identified based on a confirmed diagnosis of hyperinsulinism (HI), a history of perinatal stress (as defined in Table 1), and evidence of resolution of disease. The diagnosis of HI was established using standardized diagnostic criteria at time of hypoglycemia [plasma glucose <50 mg/dl (2.8 mmol/l)]: hypoketonemia (plasma ß-hydroxybutyrate <1.8 mmol/l) + hypofattyacidemia (plasma free fatty acids <1.7 mmol/L), if available, and/or inappropriate glycemic response to 1 mg IV glucagon [rise in glucose greater than 30 mg/dl (1.7 mmol/L) over 40 min], and/or hyperinsulinemia (plasma insulin above the limit of detection of the assay) (10). Evidence of spontaneous resolution of HI was defined as fasting greater than 12 h with plasma glucose > 70 mg/dl (3.9 mmol/L) and/or BOHB ≥ 1.8 mmol/L off of treatments for hyperinsulinism. Exclusion criteria included patients with congenital HI due to pathogenic genetic mutations, syndromic HI (such as Beckwith-Wiedemann syndrome), persistence of HI beyond age 1, patients who underwent pancreatectomy, and patients born prior to 32 weeks gestational age. Phase I of the study included a comprehensive medical record review. Subjects who were at least 1 year and less than 6 years at time of assessment (based on constraints of the instruments used) were eligible for neurodevelopmental assessments (Phase II). Subjects with congenital syndromes known or suspected to affect neurodevelopment and those with non-English speaking caregivers were excluded from developmental assessment.

**Table 1:** Subject characteristics (*n* = 107).

Demographics	*N* (%)
Male gender	80 (75)
Insurance Carrier	
Private	44 (41)
Medicaid	54 (51)
Other	9 (8)
Race & Ethnicity	
American Indian/Alaska Native	4 (4)
Asian	2 (2)
Non Hispanic Black	31 (29)
Hispanic or Latino	11 (10)
Non Hispanic White	48 (45)
Other	13 (12)
Delivery	
Prematurity^a^	38 (36)
Mode of Delivery	
Urgent C-section	48 (45)
Routine C-section	14 (13)
Induced Labor	10 (9)
Assisted Delivery	5 (5)
Spontaneous Vaginal Delivery	28 (26)
Perinatal Stressor	
Urgent/Emergent Delivery	48 (45)
Intrauterine Growth Restriction	40 (37)
Prematurity (32-37 weeks)	36 (34)
Small for Gestational Age	36 (34)
Greater than Standard Resuscitation	35 (33)
Non Reassuring Fetal Heart Tracing	33 (31)
Other^b^	31 (29)
Preeclampsia	20 (19)
Meconium	19 (18)
Congenital Heart Disease	12 (11)
Gestational Diabetes	9 (8)
Gestational Hypertension	8 (7)
Placental Insufficiency	5 (5)
Chorioamnionitis	4 (4)
Birth Asphyxia	3 (3)

^a^ Prematurity defined as <37 weeks gestation.

^b^ Other perinatal insult deemed significant in the opinion of the study physicians. Ex: twin-twin transfusion syndrome, HIE, shoulder dystocia. Note subjects must have a listed perinatal stressor in addition to an “other” stressor to meet study inclusion criteria.

This study was reviewed and approved by the Children's Hospital of Philadelphia (CHOP) Institution Review Board. Chart review was done under a waiver of consent. Under waiver of documentation of consent and HIPAA authorization eligible subjects were recruited for questionnaire completion and neurodevelopmental assessments.

### Measures

Neurodevelopmental outcomes were assessed through three validated caregiver-completed instruments: the Adaptive Behavior Assessment System – Third Edition (ABAS-3), the Child Behavior Checklist (CBCL 1.5-5), and the Behavior Rating Inventory of Executive Function – Preschool Edition (BRIEF-P). The ABAS-3 assesses adaptive behavior and is available for all ages (11). The General Adaptive Composite (GAC) score is the main outcome score of the ABAS-3 and has a mean of 100 with standard deviation (SD) of 15. Lower scores indicate worse outcomes. The CBCL assesses emotional and social functioning and is available for subjects who are 18 months through 5 years old (12). The Total Problem (TP) score is the main outcome score for the CBCL and has a mean of 50 with a SD of 10. Higher scores indicate worse outcomes. The BRIEF-P assesses executive function and is available for subjects who are ages 2 to 5 years old (13). The Global Executive Composite score (GEC) score is the main outcome score for the BRIEF-P and has a mean of 50 with a SD of 10. Higher scores indicate worse outcomes. In summation, these measures provide insight into a subject's current neurodevelopmental status.

### Statistical analysis

Baseline characteristics as well as various outcomes of interest were summarized by standard descriptive statistics. For comparisons of continuous variables of interest between subgroups (e.g., subjects with and without abnormal developmental testing results), *t*-tests were used to compare means of normally distributed data, and Mann–Whitney tests were used to compare medians of nonparametric outcome data. *χ*^2^ or Fisher's exact tests were used to examine associations between subgroups and categorical variables. Statistical significance was defined by a *P* < 0.05.

Scores on the neurobehavioral screening tools were considered abnormal if they were more than 1 standard deviation (SD) below the mean for GAC score or more than 1 SD above the mean for TP or GEC score. In order to examine differences in proportion of subjects with abnormal scores, GAC, GEC and TP scores were converted to z-scores, which reflect the number of standard deviations that a score is above or below the population mean. One sample z-tests of proportions were used to examine differences between the observed proportion of subjects and the expected proportions in a normal distribution.

## Results

### Subjects

**Figure 1:**
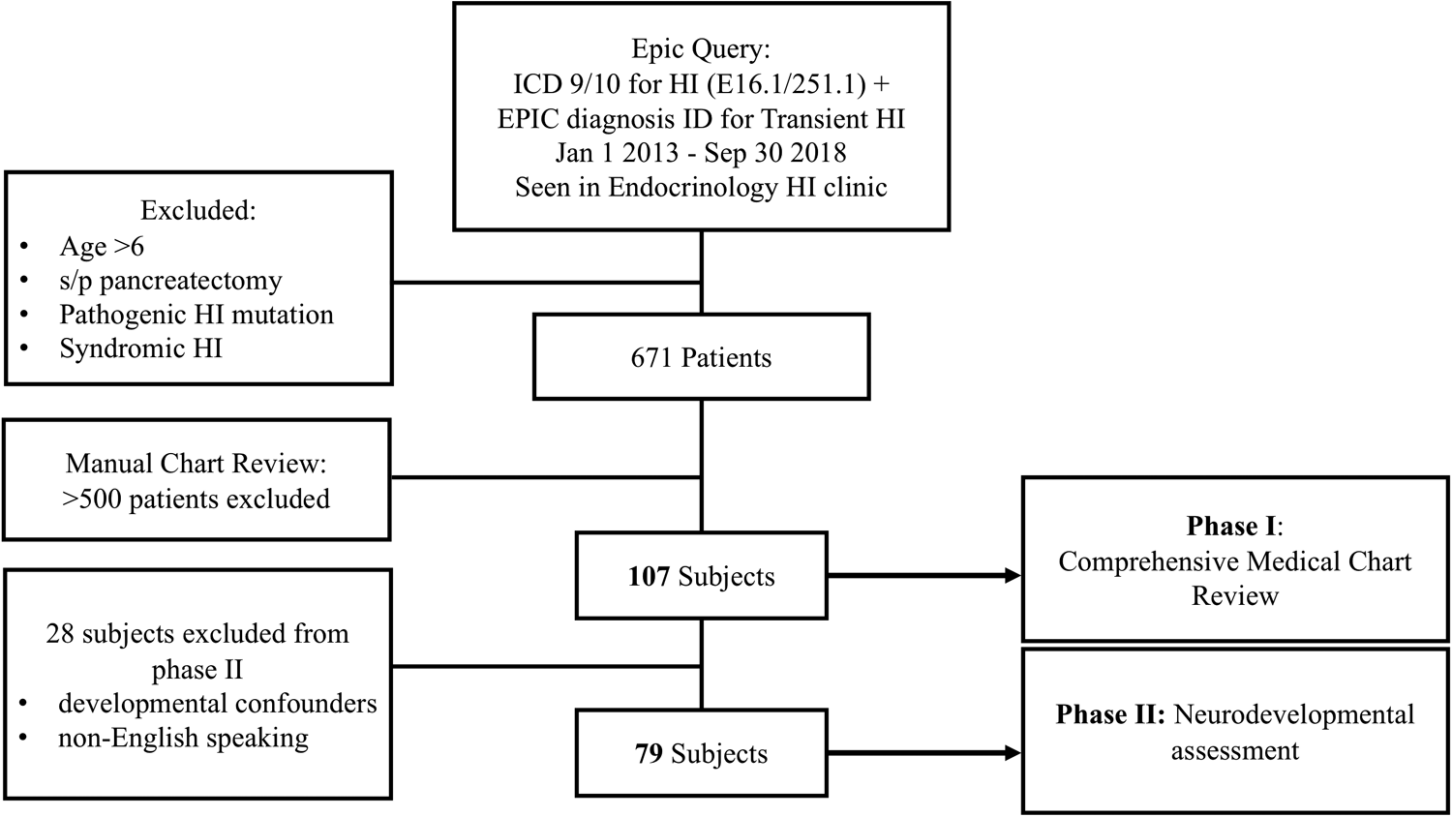
Subject identification and recruitment.

One hundred and seven subjects with a history of PSIHI, who were treated at the Children's Hospital of Philadelphia between January 2013 and September 2018, met inclusion criteria, and were enrolled in phase I or comprehensive medical chart review. Twenty-eight of those subjects did not meet criteria for phase II (neurodevelopmental assessment), due to potential developmental confounders or non-English speaking caregivers, and were excluded. Of the seventy-nine eligible subjects for phase II, twenty-three were unable to be contacted and three declined. The remaining fifty-three subjects were consented, enrolled, and interviewed. Thirty-seven of the phase II enrolled subjects (70%) completed the neurodevelopmental screening tools (Figure 1).

### Natural history

Of the 107 subjects, 80 (75%) were male. Forty five percent of subjects were born by urgent cesarean section, with an additional 13% born by routine cesarean section. Twenty six percent of subjects were born *via* spontaneous vaginal delivery. Thirty four percent of subjects were born premature at less than 37 weeks gestational age (Table 1). Mean and median gestational age was 37 3/7 weeks. Median birth weight for gestational age Z score was −1.3. Median Apgar scores at one and five minutes were 8 and 9, respectively (Tables 2).

**Table 2:** Clinical characteristics (*n* = 107).

	Mean	SD	Median	Min	Max
Gestational Age (weeks)	37 3/7	2.3	37 3/7	32	41 6/7
Birth Weight (kg)	2.56	0.8	2.56	0.8	4.99
BW for GA z-score	−1.1	1.4	−1.3	−4	3
Apgar (1 min)	6.5	2.6	8	1	9
Apgar (5 min)	8	1.8	9	1	9
Age at hypoglycemia presentation^a^ (days)			0	0	60
Age at HI Diagnosis (days)	19	20.4	12	0	141
Maximum GIR (mg/kg/min)	11.8	6.2	10.2	2	33
Time to Definitive Treatment (days)	19	16	14	0	91
Time to Cure Demonstration (days)	235	134	210	23	694

^a^ Hypoglycemia defined as glucose <2.8 mmol/L (50 mg/dl).

The subjects' clinical manifestations of perinatal stress varied, and many subjects had more than one qualifier for perinatal stress (Table 1). The most frequently met criterion of perinatal stress was an urgent delivery, in 45% of subjects. This was followed by intrauterine growth restriction, prematurity, and small for gestational age status at 37%, 34%, and 34% of subjects, respectively.

The median age at which subjects were noted to have hypoglycemia [glucose < 50 mg/dl (2.8 mmol/L)] was on day of life zero. With the exception of a few outliers, almost all subjects were noted to have hypoglycemia on day of life zero. Intravenous dextrose was used to treat 86% of subjects, with a mean maximum GIR of 11.8 mg/kg/min (median 10.2 mg/kg/min). Seven percent of subjects were treated with an intravenous glucagon infusion. Continuous feeds were used to manage 16% of patients, and 38% of patients were managed with frequent feeds. The median age at diagnosis of HI was 12 days old, and the median age at which hypoglycemic events no longer occurred was 14 days old. Seventy nine percent of subjects were successfully treated with diazoxide, and 10% were discharged on a continuous infusion of dextrose 20% through a nasogastric or gastric tube. Only two subjects discharged on a continuous enteral infusion of dextrose 20% had failed treatment with diazoxide; the remainder of these subjects experienced diazoxide side effects or had underlying risk factors for fluid overload, necessitating alternative treatment. The median time to demonstration of cure on formal fasting assessment was 210 days, or 7 months (Table 2).

Genetic testing for congenital hyperinsulinism was obtained in 81% of subjects. The type of testing varied based on the date sent, institution sending testing, and the lab performing the testing. The majority of patients had a gene panel sent that included the genes *ABCC8, KCNJ11, GCK, GLUD1, HADH, HNF4A, SLC16A1, UCP2* and *SCHAD.* All patients tested had at minimum the genes *ABCC8, KCNJ11, GCK,* and *GLUD1* evaluated. Few subjects also were tested for changes in *KMT2D* and *KDM6A*, the genes implicated in Kabuki Syndrome, as well as the epigenetic and genetic alterations of chromosome 11p15 implicated in Beckwith-Wiedemann Syndrome. No disease-causing variants were identified in any subjects.

### Neurodevelopmental outcomes

Mean subject age at time of caregiver interview was 3.7 years and 22% of subjects were female. Fifty one percent of caregivers reported concerns about the subject's development (Table 3). Similarly, approximately half (51%) of subjects had received a clinical developmental assessment prior to study enrollment. Speech delay (40%) and behavioral concerns (42%) were the most frequently reported diagnoses. Attention deficit and hyperactivity disorder (ADHD), autism, and seizures were reported at 8% each. Forty percent of patients had received speech therapy. Approximately one fourth of patients had received physical therapy or occupational therapy, at 28 and 25 percent respectively. Seventeen percent of interviewed subjects had an individualized education plan (IEP) in place or planned for the upcoming year at time of assessment.

**Table 3:** Neurodevelopment: parent interview responses (*n* = 53).

Reported Diagnoses	*N* (%)	Therapies Received	*N* (%)
Speech Delay	21 (40)	Speech Therapy	21 (40)
Learning Differences or Disability	14 (26)	Physical Therapy	11 (28)
ADHD	4 (8)	Occupational Therapy	13 (25)
Behavioral Issues	22 (42)	Special Instruction (IEP)	9 (17)
Autism	4 (8)		
Physical Disability or constraints	5 (9)		
Seizures	4 (8)		

Forty-seven percent (37 of 79) of eligible subjects' caregivers completed the ABAS-3. The median age was 4.1 years (mean age 3.9 years), and 22% were female. The mean GAC score was 98.3 ± 15.9 (Table 4). The proportion of subjects scoring more than 1 standard deviation (SD) below the mean was not significantly greater than in the general population, however, the proportion scoring more than 2 SD below the mean was significantly greater than the general population (8.3% vs. 2.2%, *P* ≤ 0.01).

**Table 4:** Neurodevelopmental assessment results.

	Mean (SD)	% >1 SD	% >2 SD
Adaptive Behavior Assessment System – III^a^ (*n* = 37)			
General adaptive composite score	98.3 (15.9)	13.9	8.3*
Conceptual composite score	97.8 (15.2)	22.2	2.8
Social composite score	99.8 (16.7)	21.6	10.8**
Practical composite score	99.4 (15.4)	13.9	8.3*
Child Behavior Checklist (*n* = 34)			
Total Problem Score	45.8 (13.3)	11.8	8.8***
Internalizing problems	44.7 (13.4)	11.8	5.9
Externalizing problems	47.5 (13.4)	8.8	5.9
Behavior Rating Inventory of Executive Functions – Preschool Edition^b^ (*n* = 31)			
Global Executive Composite	48.1 (14.9)	9.7	6.5
Flexibility Index	48.5 (14.6)	9.7	6.5
Emergent Metagognition Index	47.5 (13)	6.5	3.2
Inhibitory Self Control Index	47.4 (13.8)	16.1	6.5

^a^ Normal population mean is 100 with SD of 15; higher scores are more favorable.

^b^ Normal population mean is 50 with SD of 10; lower scores are more favorable.

* *P* < 0.01 compared to the general population.

** *P* < 0.0004 compared to the general population.

*** *P* < 0.006 compared to the general population.

Forty-three percent (34 of 79) of subjects' caregivers completed the CBCL. The median age was 4.1 years (mean age 4.0 years), and 21% were female. The mean total problem (TP) score was 45.8 ± 13.3 (Table 4). The proportion of subjects scoring more than 1 SD above the mean was not significantly greater than in the general population, however the proportion scoring more than 2 SD above the mean was significantly greater than the general population (8.8% vs. 2.2%, *P* ≤ 0.006).

Thirty-nine percent (31 of 79) of subjects' caregivers completed the BRIEF-P. The median age was 4.5 years (mean age 4.3 years), and 19% were female. The GEC score was 48.1 ± 14.9 (Table 4). The proportion of subjects scoring more than 1 and 2 SD above the mean were not significantly greater than in the general population.

The proportion of subjects scoring more than 1 SD away from the mean in the direction of concern on any of the assessment's major composite scores was significantly greater than in the general population (40.5% vs. 15.8%, *P* ≤ 0.0001), as was the proportion scoring more than 2 SDs away from the mean (18.9% vs. 2.2%, *P* ≤ 0.0001).

### Factors associated with poor outcomes

Baseline characteristics of subjects that scored abnormally on developmental assessment (more than 1 and 2 standard deviations away from the mean in the direction of concern) were compared to subjects that scored in the normal or above average range (Table 5). Male sex, small for gestational age status, Hispanic or Latino ethnicity, and treatment with continuous feeds were all strongly associated with scores greater than 1 standard deviation away from the mean in the direction of concern (*P* < 0.05) on developmental assessment. Small for gestational age status, maternal history of preeclampsia, and Hispanic or Latino ethnicity were strongly associated with scores greater than 2 standard deviations away from the mean in the direction of concern (*P* < 0.05) on developmental assessment.

**Table 5:** Association of baseline characteristics with abnormal development (*n* = 37).

Variable	Chi-Square
Association of baseline characteristics with abnormal development (>1 SD)	
Sex: Male	0.0394
Hispanic or Latino	0.0166
Race: White	0.0475
Perinatal Stress: SGA	0.0129
Treatments: Continuous feeds	0.0315
Association of baseline characteristics with abnormal development (>2 SD)	
Hispanic or Latino	0.0042
Perinatal Stress: preeclampsia	0.0462
Perinatal Stress: SGA	0.0073

When comparing cohorts of patients who scored more than 1 and 2 standard deviations away from the mean in the direction of concern to those who scored in the normal to above average range on testing, there was a trend toward lower birth weight Z score, higher GIR requirements, and longer time to diagnosis (Table 6). Despite the trends noted that suggest that low birth weight, more severe hypoglycemia, and delayed diagnosis correlate with poorer outcomes, these differences were not statistically significant.

**Table 6:** Baseline characteristics by performance on developmental assessment.

	Whole study population (*n* = 107)	Subjects who scored in normal range (*n* = 22)	Subjects who scored > 1SD (*n* = 15)	Subjects who scored > 2SD (*n* = 7)
Mean Gestational Age	37 3/7	37 6/7	37	36 6/7
% Male	75%	63%	93%	86%
Median BW for GA Z-score	−1.32	−1.09	−1.4	−1.58
Median Apgars (1,5 min)	8, 9	8, 9	7, 9	7, 9
Mean Max GIR (mg/kg/min)	11.8	8.9	13.6	17.2
Mean age at diagnosis (days)	19	14	20	24
Mean time to treatment (days)	19	14	20	16
Mean time to cure demonstration (days)	235	213	256	262

## Discussion

Perinatal stress is associated with a distinct form of transient HI (PSIHI) that often responds to diazoxide and spontaneously resolves. The mechanism of PSIHI has not been clearly demonstrated but it has been postulated that perinatal hypo-oxygenation results in delayed beta cell maturation leading to a prolongation of the physiologic transitional hyperinsulinemic state that is characteristic during the first 48 h of life (14, 15). PSIHI is often perceived as less severe than permanent congenital HI, however, this study illustrates that children with PSIHI are also at risk for severe hypoglycemia and associated comorbidities. This is also consistent with prior studies evaluating the broader group of patients with transient hyperinsulinism (6, 16, 17).

Published reports describing the natural history of PSIHI are limited to only a few small series, with varying definitions, relaxed inclusion criteria, and often lack of evidence of spontaneous HI resolution. Hoe et al. described a cohort of 26 neonates with “prolonged neonatal hyperinsulinism”, though notably no risk factors for perinatal stress were noted in 19% of the study population (6). Kozen et al. described a cohort of 40 neonates with “transient hyperinsulinism”, which included all infants who had hypoglycemia < 47 mg/dl (2.6 mmol/L) despite a 10% dextrose infusion (17). The term transient hyperinsulinism is problematic as it is nonspecific, and often includes patients with potential unidentified genetic or syndromic etiologies of HI, as evidence of perinatal stress is not always identified. A more comprehensive characterization of the distinct population of patients with PSIHI is necessary for development of targeted treatment strategies, given this population's high risk of severe hypoglycemia and resultant developmental delays.

In this study, we describe the natural history of PSIHI. Efforts were made to focus in on this particular patient population by creating strict inclusion and exclusion criteria, thereby avoiding a more general description of a broader category of patients encapsulated by the terms “genetics negative” hyperinsulinism or “transient” hyperinsulinism. All included subjects had clear evidence of perinatal stress, a confirmed diagnosis of hyperinsulinism based on standardized criteria, and importantly, evidence of spontaneous resolution of hyperinsulinism on formal fasting assessment. It should be noted that our practice at the Congenital Hyperinsulinism Center at the Children's Hospital of Philadelphia includes sending genetic testing on all patients with a confirmed diagnosis of HI, and all patients undergo an inpatient fast to assess for resolution of disease prior to being deemed resolved. Weaning or stopping therapy within the first few weeks-months of life is actively pursued if there is a history of perinatal stress, negative genetic testing, and no ongoing hypoglycemia.

### Population characteristics

We found a strong predominance of male patients (75%) in our study subjects with PSIHI and 66% of subjects were born at term or after 37 weeks. This suggests that prematurity alone is not entirely responsible for PSIHI and the notable developmental differences, as one of the largest limitations in this study is the confounding impact of other risk factors, including prematurity, on developmental outcomes. Additionally, mean and median birth weight were within the low-normal range, indicating that extremely low birth weight infants did not comprise the majority of the population. Furthermore, the mean maximum GIR was 11.8 mg/kg/min, which suggests that patients with PSIHI have more severe hypoglycemia than many clinicians may suspect.

### Time to diagnosis

While the vast majority of subjects presented with hypoglycemia on day of life 0, a diagnosis of hyperinsulinism was not confirmed until a median of 12 days of life, and patients on average continued to have hypoglycemic events until a median of 14 days of life. It is concerning that patients with clear evidence of hypoglycemia continued to have ongoing episodes of hypoglycemia for approximately two weeks, as repeated hypoglycemic events during this critical period of brain development are strongly suspected to lead to poor developmental outcomes (18). Unfortunately this delay to establish diagnosis is not uncommon, as previous studies have shown similar time frames to diagnosis of HI (6, 9). Delays in diagnosis may be in part due to erroneous assumption that the hypoglycemia observed is related to the physiologic transitional neonatal hypoglycemia that is characteristic of the first 48 h of life, when mean plasma glucose concentrations fall in healthy newborn infants to approximately 55–60 mg/dl (3.0–3.3 mmol/L), and spontaneously resolves over the first 48–72 h of life (19).

### Neurodevelopment

The association between hypoglycemia and neurodevelopmental differences is well established. McKinlay et al. demonstrated that patients with neonatal hypoglycemia, regardless of etiology, are at increased risk for low executive function and visual motor function (18). The association between hypoglycemia and neurodevelopmental differences has also been demonstrated in prior studies looking specifically at persistent forms of congenital HI. Lord et al. found a prevalence of 48% of neurobehavioral abnormalities in patients with surgically treated congenital HI (9). Similarly, Meissner et al. found that 55% of patients with congenital HI had evidence of neurodevelopmental delay (20). These neurodevelopmental delays are thought to be due to recurrent hypoglycemic events during critical times of neonatal brain development.

Our study found that there was an increased prevalence of neurodevelopmental delays in the PSIHI population. Results of developmental assessments (ABAS-3, CBCL, and BRIEF-P) demonstrated significantly deficient performance compared to the general population. Similarly, caregiver reporting of developmental concerns on interview was high at 51%. There was a strong prevalence of speech delay, learning disability and behavioral concerns. Our finding of increased rates of developmental delay in the PSIHI population is similar to rates previously reported in patients with congenital HI. In 1976, Stanley and Baker found that 36% of patients with HI had developmental delays (21). In 2013, Avapatelle et al. reported abnormal development in 47% of subjects with persistent congenital HI and 30% of subjects with transient HI (3). Similarly in 2014 Lord et al. found that 27.5% of subjects with congenital HI had abnormal scores on the ABAS-II (9). In our study, 22.2% performed abnormally on the ABAS-3. Our study demonstrates that similar to patients with persistent forms of congenital HI, patients with PSIHI are also at increased risk of developmental delays and are likely to perform below average on caregiver completed developmental assessment. With the high rates of developmental issues noted, all children with HI should receive a complete neurodevelopmental evaluation.

Our study population was quite young, at ages 1–5 years at time of evaluation. Age at evaluation is important for detection of developmental delays, given that often larger issues do not come to light until children reach school age. Indeed, this was the case in the New Zealand studies, as developmental differences in patients with neonatal hypoglycemia were not detected at 2 years, but were present on evaluation at 4.5 years in the same subject population (18, 22, 23). In prior studies, neurodevelopmental issues were often identified in school age children only after struggling academically (9). In our population, speech delay was prevalent, likely due to the detectability in this younger age group.

### Limitations

There are several limitations to our study. It is cross sectional rather than longitudinal, and so the incidence of neurodevelopmental delays could not be determined. The young age of our study population at time of evaluation limited our ability to detect neurodevelopmental delays on assessment. We collected subjective data through caregiver interview, which is prone to bias. There was a higher rate of caregiver reporting of developmental concerns on interview compared to that detected on testing. This may be due to projection bias, or over rating a child's adaptive skills or behavior on interview. The discrepancy between caregiver report and assessment results makes determining the exact prevalence of neurodevelopmental delays difficult. Furthermore, only 37 of 53 (70%) eligible and consented subjects completed the neurodevelopmental assessments. It is possible that caregivers of children with better neurologic outcomes were more willing to participate. A larger study using direct developmental testing to assess development at an older age is necessary to determine the true prevalence of poor neurodevelopmental outcomes in this population.

Our sample size was small, mostly due to our very strict inclusion criteria to exclude other forms of HI and capture the natural history of PSIHI, and also due to difficulty with recruiting and neurodevelopmental assessment completion. Due to the small sample size, we were not able to achieve statistical power to accurately assess the association between assessment performance and hypoglycemia severity, age at diagnosis, and time to definitive treatment. However, we did see notable trends suggesting there is a correlation. A notable limitation of our study is the confounding factors associated with perinatal stress that may impact neurodevelopmental outcomes of their own. Effort was made to minimize confounding factors on neurodevelopmental assessment, however, our ability to do this was limited, and thus, we cannot assume a direct effect of hypoglycemia on these outcomes, independent of other risk factors.

Despite these limitations, our study demonstrates that patients with PSIHI have a high rate of reported neurodevelopmental concerns and are more likely to perform below average on neurodevelopmental assessment. We identified baseline characteristics that are strongly associated with below average performance. Our study also comprehensively describes the natural history of strictly defined PSIHI. The majority of PSIHI patients are male, almost half of subjects were born in an urgent delivery, and most have more than one etiology of perinatal stress.

## Conclusion

Children with PSIHI are predominantly male and most are born at term. While the majority of infants presented with hypoglycemia in the first day of life, diagnosis and definitive treatment was achieved at close to 2 weeks of life. Patients with PSIHI are at high risk of neurodevelopmental deficits and are more likely to perform below average on caregiver-completed developmental assessment.

Similar to children with focal HI who are cured after surgery, patients with PSIHI who spontaneously resolve are also shown to suffer from neurodevelopmental deficits. This strongly suggests that the initial hypoglycemic insult in the immediate neonatal period prior to HI diagnosis and treatment is a strong contributor to the poor neurodevelopmental outcomes detected years later. Thus, early diagnosis and prompt and effective treatment of hypoglycemia is critical to improve long term outcomes in children with PSIHI. Implementing standardized neurodevelopmental screening guidelines for early detection and treatment of neurodevelopmental delays is recommended to optimize developmental outcomes in this at-risk population.

## Data Availability

The original contributions presented in the study are included in the article/Supplementary Material, further inquiries can be directed to the corresponding author/s.
